# Risk of Contracting COVID-19, Personal Resources and Subjective Well-Being among Healthcare Workers: The Mediating Role of Stress and Meaning-Making

**DOI:** 10.3390/jcm10010132

**Published:** 2021-01-02

**Authors:** Dariusz Krok, Beata Zarzycka, Ewa Telka

**Affiliations:** 1 Institute of Psychology, University of Opole, 45-040 Opole, Poland; 2 Institute of Psychology, John Paul II Catholic University of Lublin, 20-950 Lublin, Poland; zarzycka@kul.lublin.pl; 3 Department of Radiotherapy, Maria Sklodowska-Curie National Research Institute of Oncology, Gliwice Branch, 44-101 Gliwice, Poland; etelka@io.gliwice.pl

**Keywords:** risk of contracting COVID-19, personal resources, meaning-making, stress, subjective well-being, healthcare workers

## Abstract

The latest research suggests that the relationships between the risk of contracting COVID-19, personal resources and subjective well-being have rather an indirect character and can include the occurrence of mediating factors related to meaning-making processes and stress experiences. Protection motivation theory offers a theoretical paradigm that enables these associations to be thoroughly investigated and understood. The current study aimed to examine the mediating roles of meaning-making and stress in the relationship of risk of contracting COVID-19 and personal resources (self-efficacy and meaning in life) with subjective well-being among healthcare workers. A total of 225 healthcare workers from hospitals, medical centres and diagnostic units completed a set of questionnaires during the first few months of the COVID-19 lockdown period (March–May 2020). The results revealed that greater self-efficacy and meaning in life were associated with higher cognitive and affective dimensions of subjective well-being, whereas a lesser risk of contracting COVID-19 was only associated with the higher affective dimension. The central finding demonstrated different mediating roles of stress and meaning-making in the relationship of risk of contracting COVID-19 and personal resources with the cognitive and affective dimensions of subjective well-being. This confirmed the applicability of meaning-oriented and stress management processes for understanding how healthcare workers’ well-being is affected during the COVID-19 pandemic.

## 1. Introduction

The global pandemic caused by the novel coronavirus (COVID-19) has noticeably affected the work conditions and well-being of healthcare workers. The potential exposure and risk of contracting COVID-19, linked with new cases and quarantine procedures being systematically introduced, are understandably higher in such places as hospitals, medical centres and diagnostic units [1]. This situation creates psychologically unique conditions that have a profound impact on the mental factors responsible for the well-being of healthcare workers. As there have not been many studies into the effects of COVID-19 on healthcare workers, examining these factors can undoubtedly contribute to both understanding the psychological mechanisms underlying the workers’ behaviour and optimizing the entire healthcare system [2].

### 1.1. Risk of Contracting COVID-19, Personal Resources, and Subjective Well-Being

Previous research examining the psychosocial effects of the 2002 outbreak of severe acute respiratory syndrome (SARS) demonstrated that emotional distress occurred in 18–57% of healthcare workers and was related to fear of contracting the virus [3,4,5]. Recent studies conducted during the COVID-19 pandemic also revealed that psychological distress was associated with fear of being infected with COVID-19 from patients [6,7]. Healthcare workers are particularly vulnerable to contagion due to their frontline contact with COVID-19-infected patients, their own health problems and insufficient personal protection equipment.

There is convincing evidence suggesting that the risk of contracting COVID-19 is negatively associated with well-being. Higher perceived risk of SARS infection was related to lower psychological adjustment and higher emotional distress in SARS survivors [8]. During the first months of the COVID-19 outbreak in China, the perceived severity of COVID-19 contributed significantly to negative emotional reactions [9] and mental health problems [10]. The risk of contracting COVID-19 was found to be associated with anxiety, depression and obsessive–compulsive symptoms among medical health workers [11]; therefore it is very likely that a person’s subjective interpretation of infection rates can relate to the cognitive and affective dimensions of subjective well-being.

The underlying mechanisms responsible for the negative effect of the risk of contracting COVID-19 on well-being may be largely related to fear and anxiety. The high level of severity and susceptibility to coronavirus can generate and intensify fear and anxiety among healthcare workers, which, in turn, will influence their mental health and work efficiency during the pandemic crisis [12]. The negative feelings can worsen healthcare workers’ mental and physical well-being by undermining their self-confidence and a sense of security. As the risk of contracting COVID-19 among healthcare workers is relatively high and also unpredictable, it can produce intense distress that significantly affects cognitive (life satisfaction) and affective (positive and negative emotions) reactions. Added to the experiences, fear and anxiety is caused by workplace stress, as working conditions in hospitals and medical centres have dramatically changed in recent months [6]. Facing new professional challenges, work overload, and long-lasting job stress due to the pandemic dynamics, healthcare workers are thus prone to prolonged stress and physical and mental exhaustion, which significantly decreases their subjective well-being.

In addition to risk perception, personal resources tend to play a significant role in forming healthcare workers’ well-being. Personal resources are generally regarded as characteristics of the self that are valued by a person and are able to improve his/her effective functioning in terms of control and impact upon the environment [13,14]. Personal resources are widely applied and examined as psychological assets within the Job Demands-Resources Model (JD-R), which has been widely used in the context of occupational health psychology [15,16]. It assumes that two main categories of characteristics influence work environments: (1) job demands—they encompass physical, social, or organizational features of the job that require continued physical and/or psychological skills or effort, and (2) job resources—they include physical, social, or organizational features of the job that enable individuals to attain work-related goals, reduce job demands and their costs, and initiate personal growth and development. Excessive job demands tend to exhaust individuals’ resources and cause the depletion of energy and health problems, while job resources, due to their motivational potential, lead to high work engagement and effective performance [17]. According to the JD-R model, job resources also buffer the effect of different job demands on mental health, improving employee positive functioning. Two personal resources-meaning-making and self-efficacy models were selected due to their relevance within the JD-R model and ability to help individuals in effective reinterpretation of life experiences and successful adaptation to work tasks. Furthermore, the risk of contracting COVID-19 is likely to increase healthcare workers’ job demands, leading to lower well-being, while meaning in life and self-efficacy may buffer the negative effects of pandemic-related job demands.

Research showed that meaning in life was positively related to subjective well-being among American hospice care workers [18] and Polish healthcare personnel [2] but negatively related to depression in Turkish healthcare workers [19]. Meaning in life could be distinguished from other personal resources and was also related to well-being [20]. By providing individuals with important goals and values, enabling them to reinterpret their life experiences and effectively directing their energies, meaning in life can noticeably influence the ways in which healthcare workers deal with stress and maintain their professional efficiency [21]. Therefore, meaning in life is very likely to influence the ways in which healthcare workers deal with stress and maintain their well-being. Another important personal factor in healthcare is self-efficacy (a person’s belief in his/her abilities to succeed in particular situations), which seems to considerably assist healthcare workers to effectively fulfil their obligations and complete work tasks. Self-efficacy has also been found to be related to the well-being of healthcare workers. Examining the careers of childhood cancer survivors, Miller et al. [22] revealed that a higher level of healthcare self-efficacy was associated with a higher quality of life. In a sample of surgical residents, Milam et al. [23] demonstrated that general self-efficacy was positively related to psychological well-being and personal accomplishment and negatively related to emotional exhaustion. Given that the COVID-19 pandemic is a prolonged stressful situation, especially for those who work in healthcare, the availability of meaning in life and self-efficacy would enable the workers to manage stress and promote well-being and positive functioning.

### 1.2. Meaning-Making and Stress as Potential Mediators

The relationships among risk of contracting COVID-19, personal resources, and subjective well-being can be better understood in light of the protection motivation theory, which highlights the importance of risk perception in human reactions and describes different ways in which people are motivated to react towards a perceived threat [24]. This theory suggests that individuals will respond to threat perception either in an adaptive or maladaptive manner, on the basis of their threat appraisal and their own ability to use self-efficacy judgements to minimize the threat. According to the theory, the perceived probability that one can become infected by COVID-19 and experience harmful consequences of the infection (risk of contracting COVID-19) and also one’s individual ability to successfully apply mitigation measures (personal resources) will influence the adaptation processes. Mediating factors involving cognitive and emotional factors related to the current situation can also influence the aforementioned relationships [13,24]. Therefore, if people are able to constructively appraise the potential threat caused by COVID-19 and draw on their personal resources related to self-efficacy and meaning, they will effectively adapt to the situation and maintain a satisfactory level of well-being through meaning-making and stress-reduction processes [2].

Empirical studies examining associations between illness perception expressed in terms of risk and danger, personal resources and well-being pointed out their frequently mediational character. Meaning-making plays an important mediating role in health-related behaviour, as it enables individuals to recognise and comprehend complex situations in order to reach consistency among beliefs and goals [21,25]. Meaning-making was found to mediate the association between self-efficacy and psychological well-being in cardiac patients [26]. Self-reflection, which is a concept involving meaning-making processes, was a mediator between self-efficacy and well-being in a sample of Dutch employees [27]. In addition, the effects of global meaning in life are enhanced through meaning-making processes (e.g., daily awareness of meaning), which increases self-regulation and positively influences well-being [28]. Taking into account the internal structure of meaning-making, which reflects cognitive activities directed at perceiving and restructuring challenging situations to achieve psychological balance, this factor is very likely to contribute to life satisfaction and positive emotions.

Another factor which can mediate associations among risk perception, personal resources and well-being is experienced stress. The Protection Motivation Theory (PMT) assumes that one’s intentions (i.e., the mediating factor) can highly depend on the person’s general emotional state [13]. Therefore, stress experienced by healthcare workers can change the relationship of risk perception and personal resources with subjective well-being by exacerbating the way in which the individuals cognitively and affectively evaluate their life. Given that healthcare workers tend to experience a high level of stress, caused by work demands, lack of predictability, and continuing threats emerging from the COVID-19 pandemic, the mediating effect of stress should be particularly evident. Previous research has confirmed this assumption. Examining risk factors and the psychological consequences associated with the COVID-19 pandemic in families, Spinelli et al. [29] found that the relationship between the parents’ perception of pandemic-related difficulties and the parents’ and their children’s well-being was mediated by stress experienced by the parents. A higher level of perceived difficulties was related to higher stress, which in turn was associated with lower well-being. Stress also served as a mediator between perceptions of illness severity and anxiety and quality of life in a clinical sample of people with Crohn’s disease [30].

In addition, stress plays a mediating role between variables closely associated with personal resources and well-being, mediating the relationship between emotional intelligence and two measures of well-being (i.e., life satisfaction and psychological well-being) in a sample of graduates [31]. The underlying process through which emotional intelligence affected well-being was largely dependent on the experience of stress. Given that healthcare personnel work in highly risky and dangerous conditions, it is plausible that both risk perception and level of stress would determine the well-being outcomes.

### 1.3. The Current Study

**Figure 1:**
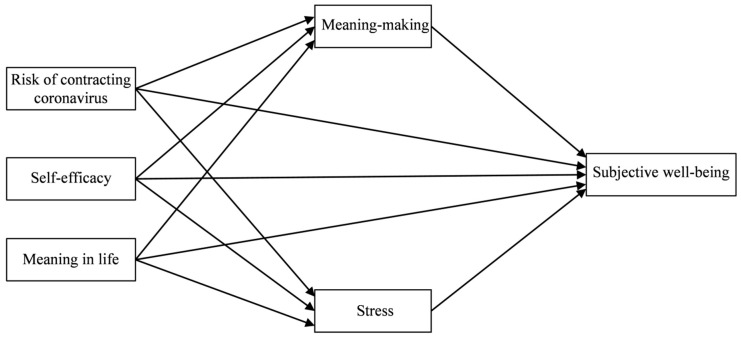
Theoretical model of the relations between risk of contracting COVID-19, self-efficacy, meaning in life, meaning-making, stress and subjective well-being.

This study aims to examine the mediating role of meaning-making and stress in the relationships between risk of contracting COVID-19, self-efficacy, meaning in life and the cognitive and affective dimensions of subjective well-being among healthcare workers. Based on the existing research presented earlier, we hypothesized that greater personal resources (self-efficacy and meaning in life) and a lesser risk of contracting COVID-19 would be associated with higher subjective well-being (Hypothesis 1). We also hypothesized that meaning-making would mediate the relationship between risk of contracting COVID-19, personal resources and subjective well-being (Hypothesis 2) and that stress would mediate that same relationship (Hypothesis 3); however, the mediational effects of meaning-making and stress should differ due to their different character. The theoretical model that includes the variables examined in the current study is shown in Figure 1.

The global pandemic caused by COVID-19 has noticeably affected the work conditions and well-being of healthcare workers. The potential exposure and risk of contracting COVID-19, linked with new cases and quarantine procedures, being systematically introduced, are understandably higher in places such as hospitals, medical centres and diagnostic units [1]. This situation creates psychologically unique conditions that have a profound impact on the mental factors responsible for the well-being of healthcare workers. As there have not been many studies into the effects of COVID-19 on healthcare workers, examining these factors can undoubtedly contribute both to understanding the psychological mechanisms underlying the workers’ behaviour and to optimizing the entire healthcare system.

## 2. Method

### 2.1. Participants and Procedure

Healthcare workers in hospitals, medical centers and diagnostic units completed a set of questionnaires during the COVID-19 lockdown period in southern Poland (March–May 2020). In order to control potential personal confounders, participants who had experienced any serious mental health problems in the previous two weeks were excluded from the study. In addition, we tried to control situational factors (i.e., the COVID-19 pandemic dynamics) by conducting our research in the shortest possible period of the initial stages of the pandemic. The quota sampling method was used to ensure the representativeness of the sample. Quota sampling was put on the five following characteristics: gender, age, types of healthcare professions, years of service, and a level of education as they thoroughly describe the group of healthcare workers in terms of sociodemographic and work features. Due to the specific features of their work, they were put at risk of contracting the virus. The study sample consisted of 225 healthcare workers: physicians (22.7%), nurses (49.8%), laboratory staff (9.8%), medical helpers (12.8%) and rehabilitation personnel (4.9%). The group comprised 126 women (58.6%) and 89 men (41.4%). The mean age of the participants was 36.18 years (SD = 13.99) and the mean years of service was 13.28 (SD = 4.37). Of the participants, 81.7% were employed full-time and 18.3% part-time. With regard to the marital status of the participants, 86.1% were married/cohabiting and 13.9% were single.

Participants were recruited in hospitals, medical centers and diagnostic units. The invitation explained that a study examining the psychological factors related to life experiences during the COVID-19 pandemic was being conducted by university academics. Interested individuals were given a set of questionnaires or provided with a link to the survey website. Research aides were available in the case of any potential questions. The participants were able to withdraw from the study at any time. All research materials and procedures were in full compliance with the university’s ethical board.

### 2.2. Measures

#### 2.2.1. Risk of Contracting COVID-19

The perceived risk of contracting COVID-19 was measured using 10 items that were scored from 1 (strongly disagree) to 5 (strongly agree) [32]. The scale was developed on the basis of “risk perception” conceptualized by Grothmann and Reusswig [33] as a measure of one’s subjective assessment of a threat’s likelihood and anticipated damage scope. Sample items are: “I am worrying that I may become infected with coronavirus” or “contracting coronavirus is dangerous to my life”. A higher score indicates a higher perceived likelihood of contracting the virus. Cronbach’s alpha coefficient for this study variable was 0.86, showing good reliability.

#### 2.2.2. Self-Efficacy

The General Self-Efficacy Scale [34] was used to assess a general sense of self-efficacy perceived by individuals in relation to their coping abilities across challenging or novel situations. Therefore, items reflect successful coping and indicate internal stable beliefs about achieving success. This widely used instrument comprises 10 items measured on a four-point scale ranging from 1 (not at all true) to 4 (all true). A higher score reflects stronger perceived self-efficacy. Cronbach’s alpha coefficient for this variable was 0.88.

#### 2.2.3. Meaning in Life

The Meaning in Life Questionnaire [35] measures two dimensions of meaning in life: presence (how much people perceive their lives in terms of meaningfulness and significance) and search (how much people strive to achieve meanings and goals in their lives). It consists of 10 items to which participants answer on a seven-point scale ranging from 1 (strongly disagree) to 7 (strongly agree). Because our study aimed at mainly tapping into the currently experienced level of meaning in life, only the presence subscale was applied. Cronbach’s alpha coefficient for this variable was 0.85.

#### 2.2.4. Meaning-Making

The Meaning-Making Questionnaire was designed to evaluate an individual’s cognitive capacity to comprehend and assimilate different or ambiguous beliefs and goals into a coherent, consistent structure that allows them to see their life as meaningful and purposeful [36]. It comprises eight items to which participants respond on a five-point scale from 1 (never) to 5 (very often). A higher score reflects a stronger intensity of meaning-making processes. Sample items are: “I focus on the activities that help me find meaning in life” or “I try to discover what is important in a particular situation”. Cronbach’s alpha coefficient for this variable was 0.87.

#### 2.2.5. Stress

The Perceived Stress Scale (PSS-10) was used to measure a level of stress experienced by individuals [37]. It contains 10 items that reflect subjective feelings related to personal problems and events, behavior and ways of dealing with them. The items are answered on a five-point scale ranging from 0 (never) to 4 (very often). Higher scores demonstrate a higher intensity of perceived stress. Cronbach’s alpha coefficient for this variable was 0.86.

#### 2.2.6. Subjective Well-Being

Two scales were used to assess cognitive and affective dimensions of subjective well-being. The Satisfaction with Life Scale (SWLS) [38] measures the extent to which individuals have an overall sense of satisfaction with life. It includes five items rated on a seven-point scale ranging from 1 (absolutely untrue) to 7 (absolutely true). Higher scores indicate a higher level of satisfaction with life. Cronbach’s alpha coefficient for this variable was 0.85. The Positive and Negative Affect Schedule (PANAS-X) [39] is a 50-item instrument that assesses the two main emotional dimensions of positive and negative affect as well as eleven more specific affects. Owing to the aim of the current study, only positive and negative affect subscales were used. They contain 20 items to which participants respond on a five-point scale ranging from 1 (very slightly or not at all) to 5 (extremely). Cronbach’s alpha coefficients for positive and negative affects were 0.83 and 0.85, respectively.

### 2.3. Statistical Analysis

*Statistica 13.1*. Dell Inc. (2016). Tulsa, OK 74104, USA and *SPSS Amos Graphics 21*. IBM Amos Development Corporation, Meadville, PA 16335, USA [40,41] were used for the analysis. First, descriptive statistics (means and standard deviations) were presented, and Pearson’s correlation coefficients were used to estimate relationships among risk of contracting COVID-19, self-efficacy, meaning in life, meaning-making, stress and the dimensions of subjective well-being. This enabled us to construct a theoretical model which encompassed the variables used in the current study. Second, path analysis was used to test the theoretical model in which we examined the mediational role of meaning-making and stress in the relationships between risk of contracting COVID-19, self-efficacy, meaning in life and subjective well-being. The analysis was conducted for both cognitive (life satisfaction) and affective (positive and negative affect) dimensions of subjective well-being, which provided the opportunity to distinguish different facets of well-being. Both direct and indirect effects were examined. Third, to examine the mediational effects of meaning-making and stress on the relationships between risk of contracting COVID-19, self-efficacy, meaning in life and the dimensions of subjective well-being, the bootstrap procedure recommended by Preacher and Hayes [42] was applied; 5000 bootstrapped samples and the 95% confidence intervals were introduced into the analysis. Standardized coefficients were used to compare the effects of the independent variables and mediators on the dependent variables.

The sample size was not determined by power analysis in advance. However, we conducted a set of post hoc power analysis. The results of the post hoc power analyses conducted on three consecutive dependent variables showed that the current sample (*n* = 225) was sufficient for effect sizes obtained in our study. In the first equation, with life satisfaction as a dependent variable, an effect size of 0.29, and *n* = 225 yielded a power of 0.98. In the second equation, with positive affect as a dependent variable, an effect size of 0.31, and *n* = 225, yielded a power of 0.99. In the third equation, with negative affect as a dependent variable, an effect size was 0.28, which, with *n* = 225, yielded a power of 0.98.

## 3. Results

### 3.1. Descriptive Statistics and Initial Correlations

The mean score for risk of contracting COVID-19 was 4.18 (*SD* = 0.63), which is slightly above the medium point of the scale. The mean ratings of self-efficacy, meaning in life, meaning-making, life satisfaction and positive affect were also above the medium point of the scale. In contrast, the mean scores of stress and negative affect were slightly below the medium point of the scale. Mean scores and standard deviations for all the scales are presented in Table 1.

Pearson correlation analysis showed that risk of contracting COVID-19 had significant positive correlations with stress and negative affect, as well as a negative correlation with positive affect. Rather surprisingly, no significant correlations were found between risk of contracting COVID-19 and both personal resources (self-efficacy and meaning in life) and meaning-making. Self-efficacy was significantly positively associated with meaning in life, meaning-making, life satisfaction and positive affect, but was negatively related to both stress and negative affect. A similar pattern was shown for meaning in life, which was positively correlated with meaning-making, life satisfaction and positive affect, but was negatively associated with stress and negative affect. Meaning-making was positively correlated with life satisfaction and positive affect, but was negatively correlated with stress. In contrast, stress was negatively related to life satisfaction and positive affect but was positively associated with negative affect.

### 3.2. Testing Mediational Relations Using Path Analysis

Path analysis demonstrated that the initial model had an unsatisfactory fit to the data: χ2 (27, *n* = 226) = 26.58; *p* < 0.001; RMSEA = 0.11; CFI = 0.84; NFI = 0.82; SRMR = 0.10. In addition, most of the direct paths were statistically nonsignificant: from risk of contracting COVID-19 to life satisfaction (*β* = −0.08, *p* < 0.172) and positive affect (*β* = −0.079, *p* < 0.239), from self-efficacy to life satisfaction (*β* = 0.05, *p* < 0.485), positive affect (*β* = 0.11, *p* < 0.064) and negative affect (*β* = −0.04, *p* < 0.513), and from meaning in life to positive (*β* = 0.06, *p* < 0.301) and negative affect (*β* = 0.04, *p* < 0.547). However, a number of indirect path coefficients turned out to be significant, allowing for a further examination of the hypothesized model.

**Figure 2:**
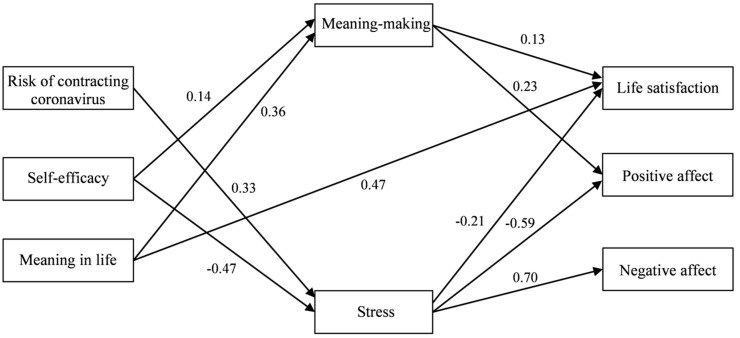
Final model of the relations between risk of contracting COVID-19, self-efficacy, meaning in life, meaning-making, stress and the dimensions of subjective well-being (all standardized coefficients are significant).

Taking into account path coefficients and modification indices, our model was retested and some nonsignificant paths were removed to optimize fit. The modifications resulted in a substantial improvement of the initial model. The final model had a good fit to the data (*χ*2 [17, *n* = 226] = 22.16; *p* < 0.001; RMSEA = 0.04; CFI = 0.95; NFI = 0.91; SRMR = 0.04) and is presented in Figure 2 (the coefficients are standardized).

In this final model, all the paths were statistically significant. There was only one direct path: from meaning in life to life satisfaction (*β* = 0.47, *p* < 0.001; 95% CI [0.35–0.58]). Risk of contracting COVID-19 was related to all three dimensions of subjective well-being (life satisfaction, positive affect and negative affect) indirectly through stress. The coefficient results indicated that higher risk was related to higher levels of stress, which again was related to lower life satisfaction and positive affect and to higher negative affect. An interesting but different pattern was found for self-efficacy, which was related to life satisfaction, positive affect and negative affect indirectly through stress, as well as to life satisfaction and positive affect indirectly through meaning-making. However, the character of their relations was different: a stronger sense of self-efficacy was related to lower levels of stress, which, in turn, was related to higher life satisfaction and positive affect and to lower negative affect. In contrast, higher self-efficacy was related to more active meaning-making, which was associated with higher life satisfaction and positive affect. Finally, meaning in life was indirectly related to two dimensions of subjective well-being (life satisfaction and positive affect) only through meaning-making. Their relationships were positive, implying that higher meaning in life was related to higher levels of life satisfaction and positive affect through more active meaning-making.

Next, the mediational effects of meaning-making and stress on the relationships between risk of contracting COVID-19, self-efficacy, meaning in life and the dimensions of subjective well-being were calculated; their results are displayed in Table 2.

The results demonstrated that none of the empirical 95% confidence intervals overlap with zero, which indicates their statistical significance. However, the mediational effects were of diverse character with regard to the particular relations between independent and dependent variables. Stress turned out to mediate the relationships between risk of contracting COVID-19 and life satisfaction, positive affect and negative affect, respectively, and between self-efficacy and negative affect. Both meaning-making and stress were mediators in the relationships between self-efficacy and life satisfaction and positive affect, respectively. In contrast, the relationships between meaning in life and life satisfaction and positive affect were mediated only through meaning-making.

## 4. Discussion

The current study aimed at investigating the mediating role played by stress and meaning-making in the associations among risk of contracting COVID-19, self-efficacy, meaning in life and subjective well-being’s dimensions among healthcare workers. To our knowledge, it is the first empirical verification of those associations in a group of healthcare personnel. The main findings revealed evident mediation through stress and meaning-making, which explains the role of meaning-oriented and stress management processes in the cognitive and affective dimensions of subjective well-being.

In support of our first hypothesis, greater self-efficacy and meaning in life were associated with higher cognitive and affective dimensions of subjective well-being, whereas lesser risk of contracting COVID-19 was only associated with the higher affective dimension. These results highlight the importance of personal resources based on meaning, purpose and beliefs of successfully completing work tasks in maintaining life satisfaction and positive emotions among healthcare workers [18,21,22]. Those healthcare workers who had clear, well-defined goals and optimistic task-related beliefs were likely to retain a satisfactory level of well-being in the face of frequent work challenges and stressful experiences caused by the pandemic. In addition, the tendency not to magnify the risk of infection was beneficial in experiencing positive emotional states. The current study also demonstrated that the risk of contracting COVID-19 was only connected to the affective dimension of the workers’ well-being (i.e., lower positive and stronger negative affect), which seems to emphasize an underlying role of affective reactions in response to pandemic threats. In line with the Job Demands-Resources Model (JD-R), the risk of contracting COVID-19 was related to higher stress caused at least partially by healthcare workers’ job demands, leading to lower well-being, while self-efficacy was related to lower stress, buffering the negative effects of pandemic-related job demands.

The central results concerned the mediating role of meaning-making and stress in the relationship of risk of contracting COVID-19 and personal resources with subjective well-being. Consistent with previous theoretical accounts [13] and empirical findings [2,27], we found that meaning-making mediated the relationship of self-efficacy and meaning in life with both the cognitive and affective dimensions of subjective well-being. The results of the path analysis demonstrated that stronger self-efficacy and meaning in life was associated with more meaning-made outcomes, which in turn was associated with healthcare personnel’s higher levels of life satisfaction and positive affect during the period of study. However, no mediation of meaning-making was found between risk of contracting COVID-19 and subjective well-being. The second hypothesis was thus partially verified.

Extending the existing literature [7,30], this pattern suggests that reinterpreting the current situation and trying to understand one’s goals and beliefs in a different way help healthcare workers to enhance their overall positive functioning. While facing adverse and risky work situations related to the pandemic, healthcare employees take action to modify their meaning systems in order to reduce their sense of discrepancy between conflicting views (e.g., dissonance between ethical obligations and feelings of fear) and restore their subjective well-being. It is also likely that the discrepancy between perceived risk of COVID-19 infection and available personal resources may prompt the employees to reorganize their global meaning systems to make the risk less aversive or reduce its threat [43]. This interpretation is supported by research conducted by Hooker et al. [28], in which meaning-making processes through regulating one’s thoughts and emotions were able to positively influence well-being.

Stress was also found to mediate the relationship of risk of contracting COVID-19 and self-efficacy with subjective well-being, which largely confirms the third hypothesis. Among healthcare personnel, higher risk was associated with higher stress, which was then related to lower life satisfaction and positive affect and to higher negative affect. In contrast, self-efficacy was associated with lower stress, which was then related to lower life satisfaction and positive affect and to higher negative affect. These findings align with previous results that revealed a mediating function of stress between the perception of pandemic threats and well-being [29] and between personal factors and well-being in young people [31]. At the same time, our findings also expand on earlier research by demonstrating that stress should be construed not only as an autonomous, physiological reaction that triggers a set of negative consequences but also as a factor that tends to work in conjunction with perceptual and dispositional factors. When estimating the amount of threat experienced, individuals consider both its severity and the available resources, which are then used to constructively cope with stress. This cognitive activity, which is based on stress management and meaning-making processes, has significant implications for well-being. The lower the stress level and the stronger the meaning-making, the higher the level of life satisfaction and positive emotions experienced by individuals.

The mediating effects of meaning in life and stress can be more deeply understood within protection motivation theory by revealing the significant role played by the person’s ability to assess the threat and use their personal resources to maintain well-being [13]. Consistent with this theory, healthcare workers’ subjective well-being during the COVID-19 pandemic depends on estimating both the risk level for contracting the virus and their motivational resources (e.g., self-efficacy and meaning in life). The relationship is likely to be influenced by various factors, among which meaning-making and stress can be found. Being in a stressful work environment severely affected by the COVID-19 pandemic, healthcare workers make attempts to maintain their well-being (i.e., protect their mental health) by appraising the threat posed by contracting the virus and, at the same time, relying on personal resources based on meaning and self-confidence. The more that workers are able to reinterpret the stressful situation to make it less aversive and reduce the stress, the higher the level of life satisfaction and positive emotions due to the more effective adaptation process [21]. In this sense, meaning-making can be considered as an adaptive cognitive activity that enables individuals to “mentally connect” the risk assessment and personal resources with subjective well-being.

Several limitations that require further discussion should be mentioned. First, we used a cross-sectional design, which does not allow causal inferences to be drawn with regard to the relations examined in the path analysis. Experimental or longitudinal designs are needed in order to establish causality among the model’s variables. Second, while measuring the affective dimensions of subjective well-being (i.e., positive and negative affect), we asked participants how they felt at the time. Although, this was a standard method for assessing the participants’ subjective well-being, emotional states are constantly changing [39]. Future research could thus attempt to measure emotions over an extended period, which may provide a more stable insight into the participants’ well-being. Lastly, the generalizability of our results can be limited due to the specific features of the Polish sample. Although we used quota sampling to ensure the representativeness of the sample, healthcare personnel in other countries can have different proportions of sociodemographic features (e.g., gender, profession, age). Future studies on attitudes towards COVID-19 and well-being among healthcare workers will therefore benefit from recruiting culturally diverse samples, especially from non-European countries.

To conclude, our study confirmed a mediating model of stress and meaning-making in the relationship between risk of contracting COVID-19, personal resources and subjective well-being in healthcare workers. Notwithstanding the above-mentioned limitations, we demonstrated that workers’ subjective well-being largely depends on the risk level of contracting COVID-19 and motivational factors, as well as the mediational processes played by stress and meaning-making. In addition, our study has some practical implications. The knowledge about the impact of risk of contracting COVID-19 on well-being could be used to make healthcare workers aware of unsubstantiated news about COVID-19 that can cause them to feel anxious or distressed. Instead, national health authorities should provide accurate scientific evidence only from trusted sources. Advanced support programs for healthcare workers that focus on personal resources (i.e., resilience) and their role in coping with stress could also be built as they are likely to protect all staff from chronic stress and poor mental health during the pandemic. Finally, psychological interventions that promote adaptive forms of meaning-making can hold the key to overcoming the fear and anxiety over COVID-19 and enhancing subjective well-being among healthcare personnel.

## Figures and Tables

**Table 1:** Means, standard deviations and correlations between risk of contracting COVID-19, self-efficacy, meaning in life, meaning-making, stress and the dimensions of subjective well-being.

	Variables	*M*	*SD*	1	2	3	4	5	6	7
1	Risk of contracting COVID-19	4.18	0.63	−						
2	Self-efficacy	3.07	0.44	0.01	−					
3	Meaning in life	5.23	1.19	0.09	0.31 ***	−				
4	Meaning-making	3.48	0.72	0.01	0.24 ***	0.40 ***	−			
5	Stress	1.92	0.64	0.30 ***	−0.45 ***	−0.20 **	−0.16 *	−		
6	Life satisfaction	4.37	0.99	−0.09	0.28 ***	0.53 ***	0.28 ***	−0.31 ***	−	
7	Positive affect	3.03	0.73	−0.21 **	0.41 ***	0.27 ***	0.30 ***	−0.61 ***	0.36 ***	−
8	Negative affect	2.23	0.77	0.29 ***	−0.33 ***	−0.16 *	−0.09	0.68 ***	−0.22 ***	−0.47 ***

* *p* < 0.05, ** *p* < 0.01 and *** *p* < 0.001.

**Table 2:** Bootstrapping-standardized indirect effects and 95% confidence intervals.

Model Pathways	Estimates	95% CI	
		Lower	Upper
Risk of contracting COVID-19 → Stress → Life satisfaction	−0.07	−0.12	−0.03
Risk of contracting COVID-19 → Stress → Positive affect	−0.20	−0.27	−0.13
Risk of contracting COVID-19 → Stress → Negative affect	0.23	0.14	0.31
Self-efficacy → Meaning-making/Stress → Life satisfaction	0.11	0.05	0.17
Self-efficacy → Meaning-making/Stress → Positive affect	0.31	0.2	0.4
Self-efficacy → Stress → Negative affect	−0.32	−0.42	−0.22
Meaning in life → Meaning-making → Life satisfaction	0.07	0.01	0.12
Meaning in life → Meaning-making → Positive affect	0.09	0.03	0.16

## Data Availability

The datasets of the present study are available in the OSF HOME repository: https://osf.io/sw8jn/.

## References

[1] Neto M.L.R., Almeida H.G., Esmeraldo J.D., Nobre C.B., Pinheiro W.R., de Oliveira C.R.T., da Costa Sousa I., Lima O.M.M.L., Lima N.N.R., Moreira M.M. (2020). When health professionals look death in the eye: The mental health of professionals who deal daily with the 2019 coronavirus outbreak. Psychiatry Res..

[2] Krok D., Zarzycka B. (2020). Risk Perception of COVID-19, Meaning-Based Resources and Psychological Well-Being amongst Healthcare Personnel: The Mediating Role of Coping. J. Clin. Med..

[3] Ho S.M.Y., Kwong-Lo R.S.Y., Mak C.W.Y., Wong J.S. (2005). Fear of severe acute respiratory syndrome (SARS) among health care workers. J. Consult. Clin. Psychol..

[4] Maunder R.G., Lancee W.J., Rourke S., Hunter J.J., Goldbloom D., Balderson K., Petryshen P., Steinberg R., Wasylenki D., Koh D. (2004). Factors Associated With the Psychological Impact of Severe Acute Respiratory Syndrome on Nurses and Other Hospital Workers in Toronto. Psychosom. Med..

[5] Tam C.W.C., Pang E.P.F., Lam L.C.W., Chiu H.F.K. (2004). Severe acute respiratory syndrome (SARS) in Hong Kong in 2003: Stress and psychological impact among frontline healthcare workers. Psychol. Med..

[6] de Pablo G.S., Vaquerizo-Serrano J., Catalan A., Arango C., Moreno C., Ferre F., Shin J.I., Sullivan S., Brondino N., Solmi M. (2020). Impact of coronavirus syndromes on physical and mental health of health care workers: Systematic review and meta-analysis. J. Affect. Disord..

[7] Shacham M., Hamama-Raz Y., Kolerman R., Mijiritsky O., Ben-Ezra M., Mijiritsky E. (2020). COVID-19 Factors and Psychological Factors Associated with Elevated Psychological Distress among Dentists and Dental Hygienists in Israel. Int. J. Environ. Res..

[8] Cheng S.K.W., Chong G.H.C., Chang S.S.Y., Wong C.W., Wong C.S.Y., Wong M.T.P., Wong K.C. (2006). Adjustment to severe acute respiratory syndrome (SARS): Roles of appraisal and post-traumatic growth. Psychol. Health.

[9] Li J.B., Yang A., Dou K., Wang L.-X., Zhang M.-C., Lin X. (2020). Chinese public’s knowledge, perceived severity, and perceived controllability of the COVID-19 and their associations with emotional and behavioural reactions, social participation, and precautionary behaviour: A national survey. BMC Public Health.

[10] Li J.B., Yang A., Dou K., Cheung R.Y.M. (2020). Self-control moderates the association between perceived severity of the coronavirus disease 2019 (COVID-19) and mental health problems among the Chinese public. Int. J. Environ. Res. Public Health.

[11] Zhang W., Wang K., Yin L., Zhao W., Xue Q., Peng M., Min B., Tian Q., Leng H., Du J. (2020). Mental Health and Psychosocial Problems of Medical Health Workers during the COVID-19 Epidemic in China. Psychother. Psychosom..

[12] Ahorsu D.K., Lin C.Y., Imani V., Saffari M., Griffiths M.D., Pakpour A.H. (2020). The fear of COVID-19 scale: Development and initial validation. Int. J. Ment. Health Addict..

[13] Floyd D.L., Prentice-Dunn S., Rogers R.W. (2000). A Meta-Analysis of Research on Protection Motivation Theory. J. Appl. Soc. Psychol..

[14] Hobfoll S.E., Johnson R.J., Ennis N., Jackson A.P. (2003). Resource loss, resource gain, and emotional outcomes among inner city women. J. Personal. Soc. Psychol..

[15] Demerouti E., Bakker A.B., Nachreiner F., Schaufeli W.B. (2001). The job demands-resources model of burnout. J. Appl. Psychol..

[16] Schaufeli W.B., Taris T.W., Bauer G.F., Hämmig O. (2014). A critical review of the job demands-resources model: Implications for improving work and health. Bridging Occupational, Organizational and Public Health.

[17] Bakker A.B., Demerouti E. (2007). The Job Demands-Resources model: State of the art. J. Manag. Psychol..

[18] Shiri S., Wexler I., Marmor A., Meiner Z., Schwartz I., Levzion Korach O., Azoulay D. (2020). Hospice Care: Hope and Meaning in Life Mediate Subjective Well-Being of Staff. Am. J. Hosp. Palliat. Care.

[19] Güngör A., Uçman A.G. (2020). Depression and hopelessness in Turkish healthcare workers: The moderating and mediating roles of meaning in life. Glob. Public Health.

[20] Reker G.T., Wong P.T., Wong P.T. (2012). Personal meaning in life and psychosocial adaptation in the later years. The Human Quest for Meaning: Theories, Research, and Applications.

[21] Park C.L. (2016). Meaning Making in the Context of Disasters: Meaning Making in the Context of Disasters. J. Clin. Psychol..

[22] Miller K.A., Wojcik K.Y., Ramirez C.N., Ritt-Olson A., Freyer D.R., Hamilton A.S., Milam J.E. (2017). Supporting long-term follow-up of young adult survivors of childhood cancer: Correlates of healthcare self-efficacy. Pediatr. Blood Cancer.

[23] Milam L.A., Cohen G.L., Mueller C., Salles A. (2019). The Relationship Between Self-Efficacy and Well-Being Among Surgical Residents. J. Surg. Educ..

[24] Westcott R., Ronan K., Bambrick H., Taylor M. (2017). Expanding protection motivation theory: Investigating an application to animal owners and emergency responders in bushfire emergencies. BMC Psychol..

[25] Lachnit I., Park C.L., George L.S. (2020). Processing and Resolving Major Life Stressors: An Examination of Meaning-Making Strategies. Cogn. Ther. Res..

[26] Krok D., Zarzycka B. (2020). Self-Efficacy and Psychological Well-Being in Cardiac Patients: Moderated Mediation by Affect and Meaning-Making. J. Psychol..

[27] Van Seggelen-Damen I., van Dam K. (2016). Self-reflection as a mediator between self-efficacy and well-being. J. Manag. Psychol..

[28] Hooker S.A., Masters K.S., Park C.L. (2018). A Meaningful Life Is a Healthy Life: A Conceptual Model Linking Meaning and Meaning Salience to Health. Rev. Gen. Psychol..

[29] Spinelli M., Lionetti F., Pastore M., Fasolo M. (2020). Parents and Children Facing the COVID-19 Outbreak in Italy.

[30] Zhang M., Hong L., Zhang T., Lin Y., Zheng S., Zhou X., Fan R., Wang Z., Zhang C., Zhong J. (2016). Illness perceptions and stress: Mediators between disease severity and psychological well-being and quality of life among patients with Crohn’s disease. Patient Prefer. Adherence.

[31] Urquijo I., Extremera N., Villa A. (2016). Emotional Intelligence, Life Satisfaction, and Psychological Well-Being in Graduates: The Mediating Effect of Perceived Stress. Appl. Res. Qual. Life.

[32] Krok D. (2020). The Risk of Contracting COVID-19 Scale.

[33] Grothmann T., Reusswig F. (2006). People at Risk of Flooding: Why Some Residents Take Precautionary Action While Others Do Not. Nat. Hazards.

[34] Schwarzer R., Jerusalem M., Weinman J., Wright S., Johnston M. (1995). Generalised Self-Efficacy Scale. Measures in Health Psychology: A user’s Portfolio. Causal and Control Beliefs.

[35] Steger M.F., Frazier P., Oishi S., Kaler M. (2006). The meaning in life questionnaire: Assessing the presence of and search for meaning in life. J. Couns. Psychol..

[36] Krok D. (2018). The Meaning-Making Questionnaire (MMQ).

[37] Cohen S., Kamarck T., Mermelstein R. (1983). A global measure of perceived stress. J. Health. Soc. Behav..

[38] Diener E., Emmons R.A., Larsen R.J., Griffin S. (1985). The Satisfaction with Life Scale. J. Personal. Assess..

[39] Watson D., Clark L.A. (1999). The PANAS-X: Manual for the Positive and Negative Affect Schedule-Expanded Form.

[40] Arbuckle J.L. (2012). IBM SPSS Amos 21.

[41] Fairchild A.J., MacKinnon D.P., Taborga M.P., Taylor A.B. (2009). R2 effect-size measures for mediation analysis. Behav. Res. Methods.

[42] Preacher K.J., Hayes A.F. (2004). SPSS and SAS procedures for estimating indirect effects in simple mediation models. Behav. Res. Methods Instrum. Comput..

[43] Park C.L. (2017). Distinctions to Promote an Integrated Perspective on Meaning: Global Meaning and Meaning-Making Processes. J. Constr. Psychol..

